# Perceived need, barriers to and facilitators of mental health care among HIV-infected PWID in Hanoi, Vietnam: a qualitative study

**DOI:** 10.1186/s12954-019-0349-8

**Published:** 2019-12-26

**Authors:** Minh X. Nguyen, Vivian F. Go, Quynh X. Bui, Bradley N. Gaynes, Brian W. Pence

**Affiliations:** 1 Department of Health Behavior, Gillings School of Global Public health, University of North Carolina, 135 Dauer Dr, Chapel Hill, NC 27599 USA; 2 Department of Psychiatry, UNC School of Medicine, University of North Carolina, Chapel Hill, USA; 3 Department of Epidemiology, Gillings School of Global Public health, University of North Carolina, Chapel Hill, USA

## Abstract

**Background:**

The HIV epidemic in Vietnam has been primarily driven by injection drug use. HIV-infected people who inject drugs (PWID) in Vietnam have very high rates of mental health problems, which can accelerate progression to AIDS and increase mortality rates. No research has explored the barriers and facilitators of mental health care for HIV-infected PWID in Vietnam.

**Methods:**

We conducted 28 in-depth interviews among HIV-infected PWID (*n* = 16), HIV and MMT (methadone maintenance treatment) providers (*n* = 8), and health officials (*n* = 4) in Hanoi. We explored participants’ perceptions of mental health disorders, and barriers and facilitators to seeking and receiving mental health care.

**Results:**

HIV-infected PWID were perceived by both PWID, HIV/MMT providers, and health officials to be vulnerable to mental health problems and to have great need for mental health care. Perceived social, physical, and economical barriers included stigma towards HIV, injection drug use, and mental illnesses; lack of awareness around mental health issues; lack of human resources, facilities and information on mental health services; and limited affordability of mental health services. Social support from family and healthcare providers was a perceived facilitator of mental health care.

**Conclusions:**

Interventions should raise self-awareness of HIV-infected PWID about common mental health problems; address social, physical, economic barriers to seeking mental health services; and increase social support for patients.

## Background

People with HIV (PWH) have very high rates of common mental health problems, such as anxiety and depression [1–5]. Mental health problems are strongly associated with negative outcomes for PWH, including worsening course of HIV [6], more rapid progression to AIDS, increased antiretroviral therapy (ART) nonadherence, and increased mortality rates [7, 8]. The specific group of PWH who are injection drug users are even more prone to these mental health problems, with up to 63% of HIV-infected people who inject drugs (PWID) meeting the criteria for depression diagnosis [3–5]. Compared to the general population of PWH, HIV-infected PWID suffer from the additional financial and emotional burdens associated with drug addiction [9, 10]. Treatment for mental health conditions is a critical step in improving HIV-infected PWID’s both emotional and physical well-being [11, 12].

The HIV epidemic in Vietnam as well as many other Asian countries is largely driven by transmission among PWID. In 2017, the HIV prevalence among PWID in Southeast Asia and Central Asia was 15% and 11%, respectively [13]. Vietnam has been identified as one of six countries accounting for half of the global population of PWID, with the HIV prevalence among this group as high as 28.5% [14]. In Vietnam, one study reported that the prevalence of moderate and severe depressive symptoms among HIV-infected PWID are 25% and 44%, respectively [15]. HIV-positive PWID are particularly vulnerable to mental health problems due to economic hardship, incarceration, HIV, and drug-related stigmatization and discrimination [10]. Despite serious consequences of mental illness on health and HIV progression, mental health issues are under-diagnosed and under-treated in people living with HIV [16].

Mental health care has received increased attention from the Vietnamese government in recent years. In Vietnam, mental health services are jointly provided by the Ministry of Health, Ministry of Education, and Ministry of Labor, Invalids and Social Affairs [17]. In 2011, the government has approved the program “Social assistance and rehabilitation projects for people with mental disorder, relying on the community 2011-2020” [17]. The National Mental Health Strategy 2016–2025 was issued by the government in 2015, with the following goals: improving mental health, preventing mental disorders, ensuring effective and equitable delivery of health and social care, protecting human rights, and reducing disease, deaths, and disabilities for people with mental disorders [18]. The strategy specified the needs to have the interdisciplinary approach to mental health care, create the National Laws of Mental health by 2020, and incorporate mental health care into preventive and community health activities. Indeed, community-based mental health programs have recently been implemented in many cities and provinces in Vietnam [17]. However, challenges to mental health treatment in the general population in Vietnam include the lack of mental health legislation, lack of human resources and psychiatric specialist facilities, and inadequate mental health services available [19, 20]. Moreover, no research has been done to explore the additional challenges in receiving mental health care among the particularly vulnerable group of HIV-infected PWID.

To inform future mental health interventions for PWID living with HIV, we conducted a qualitative research study to take a closer look at the needs for mental health care as well as the challenges of this population in getting these services. Our objective was to explore patients’, providers’, and health officials’ perspectives around the causes of mental health problems, and the need for barriers to and facilitators of mental health care among HIV-infected PWID in Hanoi, Vietnam.

## Methods

### Study settings and recruitment

In May 2018, we conducted 28 in-depth interviews among HIV-infected PWID (*n* = 16), HIV and MMT providers (*n* = 8), and health officials (*n* = 4) in Hanoi. Hanoi is the capital of Vietnam and is the city with the second highest population in the country. The prevalence of HIV among PWID in Hanoi was 25.6% and was among the highest in Vietnam, according to national data collected in 2013 [21].

HIV-positive PWID were recruited through flyers and referral from health providers at four clinics providing HIV and MMT care in different districts of Hanoi: Nam Tu Liem, Dong Da, Long Bien, and Hoang Mai. HIV-positive PWID participants who were at least 18 years old, were currently injecting drugs or with a history of injection drug use, had a confirmed HIV diagnosis, and were receiving medical care at one of these four chosen clinics were eligible to participate in the study. HIV/MMT providers were recruited in person at these clinics. Inclusion criteria for providers included being at least 18 years old and currently working at the clinic as a physician. At each clinic, four HIV-infected PWID, one HIV provider, and one MMT provider were interviewed. In addition, we also identified four health officials who were key stakeholders in the field of HIV prevention and psychiatry in Hanoi and invited them to participate in the study. After eligibility screening in a private interview room, the interviewers began the oral consent process with participants. Eligible participants who gave consent to participate were recruited into the study. The study received ethical approval from the Institutional Review Boards at the University of North Carolina at Chapel Hill (UNC) and Vietnam National Institute of Hygiene and Epidemiology.

### Sociodemographic characteristics of participants

The sample included 16 HIV-infected PWID, 12 HIV and MMT providers, and 4 health officials. Four health officials interviewed in this study included one psychiatrist, one leader at Hanoi Department of Health, one leader at Hanoi Center of Disease Control, and one official at Vietnam Authority of HIV/AIDS. Since health providers and health officials had very similar perceptions of mental health disorders and understandings of barriers and facilitators to mental health care for HIV-infected PWID, we presented their points of view together under one single group, referred to hereafter as stakeholders.

Among PWID participants, all but one was male, and the mean age was 41.5 (range 28–51). More than half of PWID participants only completed an elementary education, while the remaining had a high school education. Almost 50% of PWID were unemployed (43.8%). As for stakeholders, two thirds were male, and the mean age was 44.1 (range 29–59). The majority of stakeholder participants had a graduate degree (58.2%).

### Data collection

A semi-structured interview guide was developed by the research team and modified to be more suitable for each type of participant. To make sure the questions were culturally appropriate, the interview guide was then reviewed by Vietnamese staff at the UNC Vietnam office, who have extensive experience working with HIV-infected PWID in the past 10 years. Questions focused on exploring participants’ understanding of mental health disorders, perceptions of the need for mental health services, and barriers to and facilitators of mental health care among PWID living with HIV in Vietnam. All interviews were conducted in Vietnamese by two Vietnamese researchers who were trained in qualitative interviewing and had extensive experience in HIV research among PWID. Each interview lasted from 45 to 70 min and was conducted in a private room at the clinic (for patients and providers) or at the health official’s office (for health officials) to ensure privacy and confidentiality. We then audio recorded, transcribed, and translated the interviews into English. Interviewers also took notes of participants’ nonverbal impressions and other contextual observations. After the interviews, each participant received an equivalent of 21 USD as compensation for their time and travel.

### Data analysis

All transcripts were imported into Dedoose software for the purpose of coding and data analysis. Memos of emergent themes and patterns were written for each interview. A codebook was developed based on the main topics explored in the interview guide and common themes across all interviews. Two investigators co-coded the first 10% of the interviews, compared the results with each other, and discussed any discrepancies to ensure intercoder reliability. We refined codes’ definitions multiple times during the process of analysis, and the application of codes was updated accordingly. A qualitative matrix was created to document similarities and differences in the perceptions of mental health among PWID, providers, and health officials. Study results were generated after reviewing all memos, the codebook, and matrix.

## Results

### Perceptions of mental health disorders and depression

Interviewers asked all participants whether they had heard of the term “rối loạn sức khỏe tâm thần” (Vietnamese word for “mental health disorders”) before and if they had, what came to their minds when they heard that term. While “mental health disorders” was a familiar term to stakeholders, half of PWID participants had never heard of it before. PWID participants who had heard the term described “mental health disorders” vaguely as deterioration in health, unstable mood, or being easily irritated. A few others had heard of the term but did not know what it meant or associated “mental health disorders” with neurological illnesses. Anger was a particularly common theme brought up by interviewees when hearing the term “mental health disorders”, with five PWID and two stakeholders discussing anger and difficulties controlling one’s emotions as a manifestation of these disorders.

Participants were also asked if they have heard of the term “trầm cảm” (Vietnamese word for “depression”). Depression was a much more familiar term, with all participants including PWID having heard of depression before. Compared to PWID, stakeholders provided more detailed descriptions of depression:

In my opinion depression is a disease that involves emotional disorders. The patient usually has excessive pessimism. It is negative, and it can lead to incorrect behaviors, like they become isolated from the society, or they might have some tendencies, like committing suicide. That can happen. (33 year-old male stakeholder)

When asked what came to their minds when they heard of the term “depression,” PWIDs mostly described perceived symptoms of depression. These symptoms could either be participants’ own experience or what they had observed from people around them. Loneliness, sadness, self-pity, insecurity, and suicidal thoughts were all common feelings used by participants to describe depression. Nine out of 16 PWID said that they had depressive symptoms in the past, and these participants had the most to say about this topic. PWID who thought they had had depression symptoms before tended to be younger and have an HIV diagnosis more recently than PWID who did not. Some PWID participants believed that they were not depressed, even when they had had depressive symptoms such as sadness and social isolation in the past and could clearly describe their feelings. The more these patients talked about their symptoms, the more they began to open up with more details to share with the interviewer.

Every day it was just a circle of finding money, buying drugs, using drugs, and staying inside the house. The next day was the same. I could not escape that circle. When I went out, I saw other people who have a wife and kids, they went to work, they had a job…But me, I only had those things to do. I could not go anywhere, I did not work. It was such a disappointment. (41-year-old male PWID)

According to both PWID and stakeholder participants, depressed people also avoided all types of interaction with those around them, preferring to be alone and isolating themselves from others. People with depression were perceived to keep their thoughts to themselves, rarely talking to anyone unless they had to. For example, a stakeholder described patients with depressed symptoms:

For example, when I talked to them, they seemed detached, it was like they didn’t want to make contact, there was something about them that they wanted to isolate themselves, they were not in a happy, cheerful spirit. They seemed detached. (59-year-old female stakeholder)

### Perceived causes of mental health disorders and depression among HIV-infected PWID

Numerous problems were listed as perceived causes of mental health disorders including depression by both PWID and stakeholder participants. HIV infection and drug addiction were the two causes that were mentioned most often in the interviews. Indeed, some PWID described positive HIV status as a death sentence, believing that they would die very soon and that they no longer had a future with HIV infection. These negative thoughts were perceived to be ongoing chronic stressors that made HIV-infected PWID more vulnerable to depression. Most PWID participants mentioned one or a few HIV-related stressors, including deteriorating health, financial difficulties due to the reduced ability to work, fear of transmitting the disease to their sexual partners, and worries about their children’s future. Some PWID participants also talked about the chronic stress of not being able to share their concerns regarding their HIV infection to anyone due to the fear of stigma and discrimination:

The problems…it’s just that when they have these questions, they don’t know whom to ask, to share with. So they keep these questions in their mind, in their imagination, questions like “I have this disease, should I tell others When I tell others I have HIV, will they hang out with me anymore?” Those are the feelings that keep them wondering, gnawing at them, but they can’t tell others. (28-year-old male PWID)

Similarly, drug addiction was perceived as a significant cause of mental health problems and depression among PWID by both PWID and stakeholder participants. According to many PWID and stakeholders, society frequently associated injection drug use with criminal behaviors and had generally negative attitudes towards drug users. Experience with drug user-related stigma over time was perceived to lead to shame, low self-esteem, and insecurity, which were talked about as normal and unavoidable feelings associated with HIV infection and injection drug use. One PWID participant described his experience with stigma as an injection drug user:

People treat me differently, for example, before when I was not addicted, I went to people's houses, then people could go out, for example, people did not pay attention to me. But after I was addicted, people went out, for example, if they just had a phone, or a wallet on the table, they made sure to put it in the closet... (46-year-old male PWID)

Many PWID and stakeholder participants agreed that PWID had a constant urge to spend a lot of money on drugs, so they had more financial burdens than PWH who did not use drugs. A few participants added that some PWID who were involved in illegal activities had the additional fear of being caught by the police. Many PWID participants and even some stakeholders did not distinguish between toxic effects of substance abuse and real signs of mental health disorders, describing hallucinations and behaviors caused by using drugs as symptoms of a mental illness. There were no differences in the number of years of experience working in the field between stakeholders who did and did not make this distinction.

When they use synthetic drugs, in the first stage they have a variety of manifestations called hyperactivity, like increasing their activities. In the next stage, they start having some manifestations of depression, like “ngáo” [feeling high]. Then they have abnormal manifestations such as scratching their faces or other unusual manifestations. (34-year-old female stakeholder)

Overall, many PWID and stakeholder participants perceived the group of HIV-infected PWID as having very high risk of depression, much higher than that of the general population. There was only one provider who stated the opposite opinion, explaining that rather than being depressed, drug users were usually “ngáo” (feeling high) and talkative as a consequence using drugs.

### Perceived needs of mental health care and support

The majority of participants, both stakeholders and PWID, believed that mental health care was very important for HIV-infected PWID. Age of the participants and number of years working in the field of stakeholders did not seem to affect their opinions on the perceived needs of mental health care for PWH and PWID. Perceived consequences of mental health problems included failure to adhere to HIV and MMT treatment, poor mental and physical health, troubles with personal relationships, reduced quality of life, and death resulting from suicidal attempt. Mental health care was perceived to play a potentially critical role in helping depressed patients prevent the serious consequences of depression and resume to their normal life. Having stable mental health and positive thoughts would allow patients to take better care of themselves and give patients the motivation to overcome many difficulties they were facing in life. Many PWID and stakeholder participants explained that families and the society would also benefit from these changes, since mentally stable patients would need less help in everyday activities and were less likely to do harmful things to themselves and people around them.

I mean people with mental illness, I know ... they can be dirty, they refuse to take a bath ...do not listen to their family. In such a situation, when patients are treated and they return to the normal life, they will take bath regularly, there will be less work for their family to take care for them, their family does not need to spend time on that (52-year-old female stakeholder)

Both PWID and stakeholder participants felt that social support was critical for individuals suffering from depression and other mental health problems. Important sources of support mentioned included family and friends, HIV and MMT providers, counselors, and mental health specialists, such as psychiatrists. All PWID and stakeholder participants agreed that mental health care and support from family and health providers were both necessary, except for one PWID who believed that family support was the single most important factor:

I don’t think that he should see a doctor for depression. I simply think that if someone has depression, he needs supports from his family first. I don’t think about doctors. Simply, depression is a shock of feelings, mostly because of love so I think it needs love from family. I don’t think about doctors. (35-year-old female PWID)

All participants were asked about their perceptions of two different approaches to mental health treatment: using medications and psychological counseling. PWID and stakeholders perceived the relative importance of these two approaches differently. The majority of PWID and stakeholder participants thought that both approaches are effective and a combination of using medications and therapy might be the best strategy to treating mental health disorders. However, a few PWID did not think using medications was effective in treating mental disorders including depression. Other PWID did not dismiss the importance of medications but believed that psychological counseling was much more important. One PWID and one stakeholder talked about potential issues with using medications to treat depression, such as interactions with HIV drugs and methadone, adherence as well as increased costs.

### Perceived barriers to mental health treatment

#### Denial of mental health problems

Denial of mental health problems including depression emerged as a barrier to mental health seeking behavior among HIV-infected PWID. This lack of awareness was mentioned more often by stakeholders than by PWID themselves, especially HIV providers who had daily contact with patients. Some providers explained that since patients were rarely aware of their problems, other people around them helped them seek mental health care. According to many stakeholders, patients were “confused,” or even “irritated” when they were referred to mental health facilities, believing that they were mentally fine and did not need treatment. This could be in part the belief that mental health problems only included the most severe forms of mental illness such as psychosis or violence, not moderate depression or anxiety. Many PWID had symptoms of depression but did not consider themselves having a mental issue, and the limited definition of mental health prevented them from feeling they needed care as illustrated by this quote by one PWID:

I was not crazy, it was not a disease yet for me to go to the asylum. It was just that I had dark thoughts and avoided other people. I think it was hard to say “I need treatment for my depression”, because no one can examine me for that disease. And I was not that crazy to go the asylum. That’s difficult. (44-year-old male PWID)

#### HIV-related stigma and mental health-related stigma

Almost half of all participants, including both PWID and stakeholders mentioned HIV or mental health stigma as a challenge in seeking help for mental illnesses at medical facilities.

First, PWID who experienced HIV-related stigma believed that health providers easily changed their attitudes once they knew the patients were HIV infected. These participants perceived that many providers tended to avoid patients, refused to provide treatment, made them wait for longer period of time, and wore additional gloves to protect themselves from HIV. Experiences with HIV stigma in the past were perceived to lead to insecure feelings and low self-esteem among these participants, who wanted to avoid interaction with medical providers as much as possible. For example, one PWID participant explained:

Every time I went to see doctors, I went to different hospitals each time. When I was there for an examination, I got tested… I did not hide my name, my age. And at the hospital when they tested me they found out. There might be people that I know at some of the hospitals, so they would accidentally know about my HIV infection. And the information could spread, that was why I felt insecure. (45-year-old male PWID)

Stakeholders participants also agreed that such provider HIV-related stigma existed, with 6 out of 12 stakeholders listing HIV stigma as a barrier to seeking mental health care. Stakeholders who did were more likely to be female and older compared to those who did not.

More than one third of participants, including both PWID and stakeholders, mentioned mental health-related stigma as a barrier to uptake of mental health services. Specifically, fear of lack of confidentiality and being labeled as crazy or abnormal, which in turn would have a negative impact on their family prevented patients from seeking mental health care.

Well, mental illnesses, as far as I know, there is a fear in the mind, if in a family there is a crazy person, for example, that is, there might be genetic factors involved. Everyone is aware that this family has a person like that, so without adequate knowledge, people might think the disease is transmitted from one generation to the next. So people think, for example ... when getting married, people also consider that. Because of that, patients would rather be treated at other places, or depending on the degree of their disease they need to be treated in higher level facilities, for example, they can travel a far distance. (52 year-old female stakeholder)

#### Lack of human resources, facilities and information on mental health services

More than half of PWID participants and almost all stakeholders noted the lack of mental health facilities and human resources as barriers to mental health treatment for PWH. One PWID said:

Mental health facilities…as far as I know, there is no mental health center for HIV patients, no place to treat depression for them….I think there is no such place. (28 year-old male PWID)

These participants also thought the few mental health facilities that existed were too crowded with too few psychiatrists on staff. The lack of psychiatrists was mentioned by some stakeholders, who perceived this as a national challenge due to the specialty being technically difficult, tiring working environments, and low wages compared to other types of doctors. Mental health physicians were not afforded the same status as other physicians, reflecting the stigma around mental health itself in Vietnam:

In fact, as you know, studying medicine, no one wants to study mental health. In the past, we are afraid of telling to work on mental health… People want to find another job, hot jobs such as surgery, either obstetric or cardiovascular, but the psychology was simply considered as contacting to people with mental disorders so it may affect to them, psychiatrists. (49-year-old male stakeholder)

As a result, the vast majority of both PWID and stakeholder participants also perceived the mental health care system as having low quality of care in general. However, there were two stakeholders who felt the facilities and resources for mental health care were adequate. They explained that patients having mental health illnesses only needed to come to existing mental health clinics and hospitals in Hanoi.

Moreover, both PWID and stakeholder participants agreed that the lack of information on where to access mental health services made it difficult for patients to seek treatment. When being asked where they would go for treatment if they had depression, the majority of PWID participants did not have an answer, saying they needed to ask other people about it.

#### Affordability of mental health services

Half of PWID participants mentioned the affordability of mental health services as a factor influencing the willingness to seek treatment, and the majority of these participants was unemployed. They stated that they might not want to, or have the financial resources, to pay for mental health services. Due to existing financial burdens associated with HIV infection and drug addiction, they would only consider mental health services if the costs of these services were low.

Participant (P) : If it [mental health care] is similar to expense of taking Methadone, I can afford it.

Interviewer (I) : How much is that?

P: It’s more than 200 thousand VND per month. From 200 to 300 thousand VND per month

…

I: What about if it is higher?

P: Maybe I can’t afford because we have to limit our drug intake in order to save money for the medicine. So, it is higher, we can’t afford it. (35-year-old female PWID)

Financial concerns only came up in two of the interviews with stakeholders. One stakeholder agreed with PWID participants, explained that mental healthcare was too expensive and because mental health problems were not considered life-threatening by patients, and obtaining care was not a priority. However, another stakeholder disagreed, believing that finance was unimportant when it came to seeking mental health services.

### Facilitators of mental health treatment

#### Social support

Family was seen as a very important source of support for patients with mental health problems by both PWID and stakeholder participants. About one third of all participants believed that family members played a crucial role in recognizing mental health problems and encouraging patients to seek medical support. Family members were perceived by these participants as having very important roles, such as talking to patients about their problems, identifying mental health care services, and accompanying patients to mental health clinics or supporting them financially.

Support from medical professionals to facilitate access to mental health services was mentioned by two PWID and one stakeholder. According to these participants, since HIV and MMT providers had frequent interactions with HIV-infected PWID, they could identify patients’ mental health problems, provide counseling, and guide patients to the appropriate mental health care facilities.

Depression…when an HIV patient comes to a hospital or a center for an examination, doctors are the ones who give him the best advice, like give him the direction, a clear direction so that the questions in his mind are answered. It’s like when a blood vessel becomes clogged up, you need to unclog it. (28-year-old male PWID).

## Discussion

Our study found that even though many PWID participants had symptoms of mental illnesses, especially depression, and could elaborate their feelings and thoughts when depressed, they had limited understanding of mental health disorders in general. HIV-infected PWID were perceived by both PWID and stakeholder participants as particularly vulnerable to depression and other mental health disorders, due to the double burden of HIV infection and drug addiction. A number of barriers to mental health care were identified, and social support was seen as critical to facilitating health seeking behaviors for mental health disorders.

To our knowledge, this is the first qualitative study that investigated perceptions of mental health and mental health care services among HIV-infected PWID. However, depression and anxiety disorders have consistently been shown to be common mental health problems among injection drug users living with HIV [3–5]. A study in Vietnam in 2013 revealed that among HIV-positive PWID, 44% had severe depressive symptoms, and another 25% experienced depression at a moderate level [15].Our results were in accordance with these findings, with many of our PWID participants reporting symptoms of depression and the majority of both PWID and stakeholders participants agreeing that HIV-infected PWID were at high risk for depression and other mental health disorders.

Our participants listed a number of life challenges that caused depression among HIV-infected PWID, such as HIV- and drug-related stigma, financial burdens, health deterioration, and work and relationship problems. A study by Tomori et al. did not specifically look at causes of depression but reported similar life challenges among HIV-infected PWID in Vietnam. Specifically, they also found that HIV- and drug-related stigma, financial difficulties, and increased social isolation were important factors that contributed to the daily struggles of PWID [10]. In 2012, another study among a sample of HIV-positive male PWID in Vietnam reported the strong influence of stigma related to injection drug use and HIV/AIDS on participants’ decision to disclose their HIV status [22]. Our results confirmed this dilemma, with many PWID describing a tendency to hide their HIV infection for fear of stigma and discrimination. Indeed, our participants explained that not having someone to confide in and to share one’s concerns significantly increased their risk of depression.

Perceived endogenous and exogenous barriers to mental health care among HIV-infected PWID were identified. One of the most prominent perceived barriers was self-denial of depressive and other mental health symptoms and therefore lack of perceived need for care among PWID. However, even when a PWID recognized depression and wanted to get help, there were other social, physical, and economical challenges to accessing care. A study among the general population in Canada reported that individuals with depressive and anxiety disorders had difficulties assessing mental health services because they preferred to manage themselves, did not know how and where to get help, could not afford help, or professional help was not available to them [23]. Compared to this finding, we found some similar perceived barriers but our sample had the additional challenges of HIV- and drug-related stigma and lack of perceived need for mental health services. This discrepancy could be explained by the very different characteristics of two groups, with one being HIV-infected PWID in a low-income country, and the other the general population in a developed country. According to the Vietnam Law of Drug Prevention and Control issued in 2000, drug users over 18 years old were obligated to go to detoxification centers for 1 to 2 years [24]. This law had been great barrier for PWID to reach out and seek mental health care. However, recent changes in the Vietnamese laws have abolished compulsory detention in detoxification centers: as of November 15, 2012, the new legal Decree 96/2012/ND-CP, stated that people with opiate addiction had the choice of participating in the methadone maintenance treatment (MMT) program and did not have to go to detoxification center for drug treatment [25].

Our results of perceived barriers to mental health care provide information on the solutions that could potentially facilitate the process of seeking and using mental health services. Many PWID in our sample only thought of mental health disorders as severe illnesses with psychotic symptoms. Therefore, raising awareness among HIV-infected PWID about common mental health problems and their symptoms is an important first step in helping patients accessing mental health care. In order to help HIV-infected PWID understand their own needs of mental health care and reduce stigma associated with mental health problems, we should consider expanding the perceived definition of mental disorders to include less severe forms of mental health problems such as moderate to mild depression and anxiety. Moreover, changes in health care policies that could increase medical students to specialize in psychiatrists are also needed. Interventions that integrate mental health care into existing HIV and MMT care could also help reduce stigma and increase access to mental health care for HIV-infected PWID.

Social support was perceived as a facilitator of mental health care. This finding was not surprising, since many studies have reported the important role that family played in the life of HIV-infected PWID in Vietnam [26, 27]. Therefore, we recommend that future interventions that aim to improve mental health for this population should integrate components of social support from family and friends of patients. Apart from support from family, we also found that support from medical professionals such as HIV and MMT providers and counselors could make it easier for HIV-infected PWID to assess mental health services. These findings highlight the importance of training for HIV and MMT providers, so that they have the tools and knowledge to perform initial mental health evaluation and screening for patients at the clinic.

Our study had several limitations. First, our sample might not be representative of all HIV-infected PWID and stakeholders in Hanoi, since our study had a small sample size, PWID participants were only recruited from four clinics providing HIV and MMT care and all but one of our PWID participants were male. However, we do not expect the dominance of male gender in our sample to heavily bias study findings, since the vast majority of PWID in Hanoi are male, as shown in other studies among this population [28, 29]. The second weakness of the study was that our limited ability to make inference about HIV-infected PWID in Vietnam, or in other low- and middle-income countries, since our study was conducted only among participants in Hanoi. Future research with a more geographically diverse sample of HIV-infected PWID can help increase generalizability for study findings.

## Conclusions

This is the first study to our knowledge that has looked at the barriers and facilitators of mental health services use among HIV-infected PWID, a vulnerable population with high rates of mental illnesses. We were able to explore not only the perceptions of patients but also those of HIV, MMT providers, and other health officials working in the field. Our findings shed lights on the many struggles that HIV-infected PWID faced along the way of achieving mental well-being and provided directions for future interventions and policies.

## Data Availability

The dataset generated and/or analyzed during the current study are not publicly available due to confidentiality and privacy issues but are available from the corresponding author on reasonable request, with all identifiable information already removed.

## Abbreviations

AIDS: Acquired immune deficiency syndrome
ART: Antiretroviral therapy
HIV: Human immunodeficiency virus
MMT: Methadone maintenance treatment
PWH: People with HIV
PWID: People who inject drugs
UNC: University of North Carolina
USD: United States dollars
